# Immunosuppressive adenosine-targeted biomaterials for emerging cancer immunotherapy

**DOI:** 10.3389/fimmu.2022.1012927

**Published:** 2022-10-25

**Authors:** Qi Wei, Lening Zhang, Nan Zhao, Zhihua Cheng, Hua Xin, Jianxun Ding

**Affiliations:** ^1^ Department of Thoracic Surgery, China-Japan Union Hospital of Jilin University, Changchun, China; ^2^ Key Laboratory of Polymer Ecomaterials, Changchun Institute of Applied Chemistry, Chinese Academy of Sciences, Changchun, China; ^3^ Department of Vascular Surgery, General Surgery Center, the First Hospital of Jilin University, Changchun, China

**Keywords:** adenosine, immunosuppressive microenvironment, immune cell, biomaterial, immunoregulation, cancer immunotherapy

## Abstract

Immunotherapy has paved the way for the future of cancer therapy, but there are still significant challenges to be overcome, such as the occurrence of immune escape or suppression. Adenosine is essential in modulating the immune responses of immune cells and maintaining immune tolerance. Emerging adenosine pathway inhibitors are considered a breakthrough in cancer immunotherapy, with emphasis first being placed on the top-down blockade of adenosine signaling axis, followed by combination therapy. However, these therapeutic strategies rely on adenosine inhibitors, mainly small molecules or antibody proteins, which are limited by a single route of administration and off-target toxicity. Therefore, synergistic nanomedicine with accurate delivery targeting deeper tumors is focused on in preclinical studies. This review discusses how adenosine reshapes immunosuppressive microenvironments through its effects on immune cells, including lymphocytes and myeloid cells. Additionally, it will be the first discussion of a comprehensive strategy of biomaterials in modulating the adenosine signaling pathway, including inhibition of adenosine production, inhibition of adenosine binding to immune cells, and depletion of adenosine in the microenvironments. Furthermore, biomaterials integrating multiple therapeutic modalities with adenosine blocking are also discussed as a promising strategy for promoting cancer immunotherapy.

## Introduction

As early as the last century, several landmark studies established the importance of extracellular adenosine (eADO) in broad immunosuppressive regulation (1, 2). eADO is produced primarily through the hydrolysis of adenosine triphosphate (ATP). ATP released extracellularly acts as an immunogenic signal to be phagocytosed by antigen-presenting cells (APCs) and triggers antitumor immune responses. The conversion of pro-inflammatory ATP to immunosuppressive adenosine is catalyzed by cell surface ectonucleotidases, such as CD39 and CD73 (3). Then, eADO binds to receptors on immune cells and regulates downstream cyclic adenosine 3′,5′-monophosphate (cAMP) signaling, which inhibits the proliferation, survival, activation, and effects of immune cells and mediates immune escape from tumors (4). Therefore, modulation of the adenosinergic signaling axis is key to reversing immune cell function, reshaping the immunosuppressive microenvironments, and enhancing cancer immunotherapy.

Antagonists targeting various components of adenosinergic signaling axis are now emerging and showing antitumor effects in Phase I/II clinical trials (5). These antagonists act primarily at the tumor site, requiring optimized delivery regimens that allow the drug to reach the tumor microenvironments (TMEs) and maintain activity therein, rather than being isolated in the periphery. In addition, early clinical trials have shown moderate benefit from the single-agent blockade. Considerable research is underway to combine adenosine signaling axis antagonists with other therapies (*e.g.*, chemotherapy, immune checkpoint therapy, and adoptive cell therapy) in clinical trials (5).

**Figure 1:**
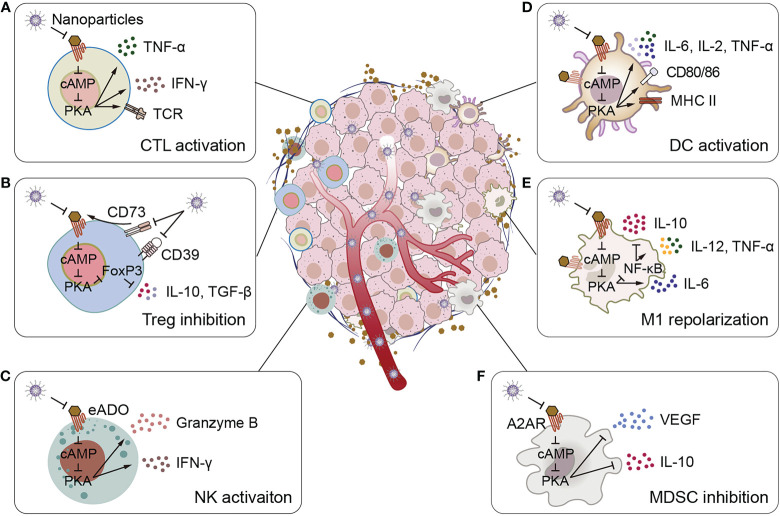
Schematic illustration of adenosine-mediated immunosuppressive TMEs reversed by biomaterials. **(A)** Biomaterials activate CTLs, promote CTLs to express TCR and secrete pro-inflammatory cytokines IFN-γ and TNF-α. **(B)** Biomaterials inhibit adenosine production and adenosine binding to receptors, thereby inhibiting IL-10 and TGF-β secretion by Tregs. **(C)** Biomaterials activate NK cells to promote the secretion of granzyme B and IFN-γ. **(D)** Biomaterials stimulate the expression of surface activation markers of DCs (CD80, CD86, and MHC II) and the secretion of pro-inflammatory cytokines IL-2, IL-6 and TNF-α. **(E)** Biomaterials promote the polarize of macrophages to M1 phenotype, inhibit the secretion of the anti-inflammatory cytokine IL-10, and promote the secretion of the pro-inflammatory cytokines IL-6, IL-12 and TNF-α. **(F)** Biomaterials inhibit MDSCs to secrete VEGF and IL-10.

**Figure 2:**
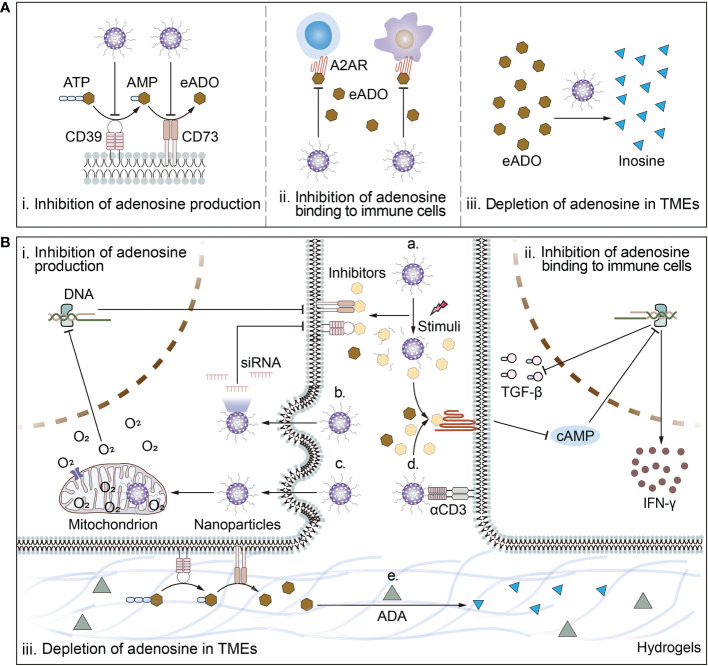
Schematic illustration of biomaterial-based strategies to target adenosine in TMEs and deep mechanisms. **(A)** Biomaterial-based strategies to target adenosine include i) inhibition of adenosine production; ii) inhibition of adenosine binding to immune cells; and iii) depletion of adenosine in TMEs. **(B)** The mechanisms of biomaterial-based strategies to target adenosine. Mechanisms by which biomaterials inhibit adenosine production include a) responsive release of inhibitors of CD39 or CD73; or b) reduction of ATP-converting enzyme receptor expression levels by delivery of siRNA for CD73 or CD39 and c) amelioration of cellular hypoxia. Mechanisms by which biomaterials inhibit adenosine binding to immune cells include a) responsive release of inhibitors of A2AR or d) surface modification of ligands targeting immune cells in close proximity to release antagonists to block the binding of adenosine to A2AR. Mechanism by which biomaterials deplete adenosine from the TMEs is e) piggybacking and releasing drugs that break down adenosine. For example, ADA breaks down adenosine into inosine.

Advanced biomaterial carrier technology is advantageous in this case. The involvement of biomaterials enables local and sustained drug delivery and multiple drugs to achieve simultaneous synergy for optimal antitumor effects, with promising medical prospects. However, there is little discussion on the role of biomaterials in regulating the adenosine signaling axis. Therefore, in this review, we mainly highlight the immunosuppressive regulation of tumor-infiltrating immune cells by eADO and the strategies of biomaterials to reverse this immunosuppression (**Figure 1**). These strategies include biomaterials assisting in inhibiting adenosine production, blocking adenosine binding to immune cells, and facilitating the conversion of adenosine to other immune activators (**Figure 2**). Rational material design is a prerequisite to achieving the strategies. First, antagonists are delivered consistently by surface modification of biomaterials to target specific immune cells (*e.g*., labeled anti-CD3 antibody targeting T cells) (6). Second, the biomaterials respond to internal and external stimuli to release antagonists to alleviate the immunosuppressive microenvironments, demonstrating extraordinary tumor regression and metastasis inhibition in murine-derived tumor models, such as the 4T1 breast cancer (7). Furthermore, direct down-regulation of enzyme receptor expression by exploiting the properties of material itself is also an option. For example, the redox ability of biomaterials is used to generate oxygen to relieve hypoxia, thus reducing adenosine production (8). In conclusion, this review provides insight into the intersection of biomaterials and adenosine-based pathways for cancer immunotherapy.

## Adenosine-mediated immunosuppressive tumor microenvironments

High eADO levels at micromolar concentrations in tumor tissues result in altered properties of cellular and biochemical components of the microenvironments compared to nanomolar levels in the physiological state. Remarkably, tumor cells and regulatory T cells (Tregs) with high expression of CD73 significantly promote eADO production in numerous tumors, such as lung cancer, breast cancer, and gastric cancer (9–11). Then, eADO acts on high-affinity adenosine 2A receptor (A2AR), often highly expressed in immune cells, to inhibit effector lymphocyte T cells and natural killer (NK) cells, and promote differentiation of CD4^+^ T cells to Tregs. eADO also activates the lower affinity adenosine 2B receptor (A2BR), expressed on myeloid cells, resulting in inhibition of dendritic cell (DC) activation and recruitment of tumor-associated macrophages (TAMs) and myeloid-derived suppressor cells (MDSCs) (12).

### Effect of adenosine on tumor-infiltrating lymphocytes

In the immunosuppressive microenvironments, CD8^+^ T cells upregulate A2AR expression and promote intracellular cAMP-PKA signaling in response to eADO activation (3). This signaling pathway dampens T cell receptor (TCR) signaling and CD28 signaling for T cell activation and the secretion of inflammatory cytokines, such as interferon-γ (IFN-γ), tumor necrosis factor (TNF), and interleukin-2 (IL-2), suppressing the cytotoxic effect on tumor cells (13, 14). Moreover, immunosuppressive regulation of adenosine is also achieved through A2AR by upregulating T cell co-inhibitory signals, including programmed cell death 1 (PD-1), cytotoxic T lymphocyte antigen, and lymphocyte activation gene 3 protein to induce T cell dysfunction (15). Knockdown of A2AR increases the levels of IFN-γ and TNF secreted by T cells (13). When A2AR blockade is combined with PD-1 inhibitors, it significantly reduces tumor progression and achieves partial or complete remission in patients with non-small cell lung cancer (NSCLC) (16). This also shows that the exhaustion of T cells through adenosinergic signaling axis is one of the mechanisms of tolerance to immune checkpoint therapy.

NK cells are one of the cytotoxic lymphocytes that inhibit tumor growth, and their activity and antitumor immune response are inhibited by eADO. Consistent with cytotoxic T lymphocytes (CTLs), eADO-A2AR-mediated signaling limits NK cell maturation. Young et al. reported that in A2AR-deficient mice, the proportion of terminally mature NK cells in the TMEs increased and maintained a stable proliferative advantage (17). Moreover, A2AR blockade also promotes the secretion of perforin, granzyme B, and interferon by NK cells, enhancing their ability to kill tumor cells (18). Furthermore, eADO in the TMEs mediates immunosuppression by increasing the expression of CD73 in NK cells (17). In conclusion, the adenosine signaling axis acts as a negative immunomodulatory signal that hinders the development of mature NK cells.

Tregs play a key role in maintaining immune homeostasis and suppressing immune responses. eADO promotes the differentiation of CD4^+^ T cells to Tregs (CD4^+^Foxp3^+^) phenotype *via* A2AR (19). In addition to tumor cells, Tregs highly expressing CD39 and CD73 are one of the main sources of eADO production in the TMEs. Moreover, Foxp3 expression shows a positive correlation with CD39 expression (20), further aggravates the immunosuppressive feature of the TMEs and promoting tumor progression by evading immune surveillance. Differences in surface markers of Tregs determine their ability to produce eADO and the mechanism of induction of immune tolerance. For example, CD39^+^ Tregs control T helper cells 17 (Th17) immune responses (21). CD39^−^CD73^−^ Tregs are unable to produce sufficient eADO and may maintain an immunosuppressive microenvironment by secreting interleukin-10 (IL-10) or transforming growth factor-β (TGF-β) (22).

### Effect of adenosine on tumor-infiltrating myeloid cells

As a bridge between innate and adaptive immunity, DCs present antigens to naive T cells to initiate an immune response. However, adenosine inhibits DC activation and antigen presentation and induces DC tolerance. Upon activation of A2AR, eADO downregulates the expression of co-stimulatory signals CD86 and major histocompatibility complex II (MHC II), and inhibits the secretion of inflammatory cytokines, such as IL-12 and TNF (1). Moreover, DCs upregulate the production of anti-inflammatory and tolerance factors, such as IL-6, IL-10, and TGF-β, in an A2BR-dependent manner (23). DCs with this tolerance phenotype fail to trigger antigen-specific CTL responses while reducing the differentiation of CD4^+^ T cells to pro-inflammatory Th1, therefore promoting tumor growth (12).

Macrophages are another type of cells of myeloid origin in the TMEs, also known as TAMs, with two main phenotypes: antitumor M1 and pro-tumor M2. Adenosine blocks the differentiation of monocytes into macrophages, eliminates the phagocytic activity of macrophages, and inhibits the secretion of inflammatory factors. For example, macrophages produce the antitumor-active factor nitric oxide (NO), but the critical inducible NO synthase is reduced by eADO (24). In addition, eADO causes M1 to M2 polarization of macrophages. Toll-like receptor signaling upregulates the expression of A2AR and A2BR, and their activation leads to tolerance and induction of the M2 phenotype (25). Moreover, high secretion of eADO in the TMEs will recruit more TAM accumulation and thus suppressing CD4^+^ T cell proliferation by increasing the expression of CD39 and CD73 (26). Therefore, inhibition of CD39 decreases eADO, increases extracellular ATP levels, and triggers the inflammatory death of tumor-promoting macrophages (27).

Furthermore, adenosine also acts on MDSCs to shape the immunosuppressive microenvironments. On the one hand, eADO specifically binds A2AR on MDSCs to mediate immune tolerance. The absence of A2AR in MDSCs was shown to significantly increase the abundance of CTLs and NK cells in the TMEs and slow tumor progression in mice with Lewis lung cancer (28). On the other hand, the activation of eADO-A2BR signaling suppresses the immune response in a manner that amplifies the pro-tumor Gr1^hi^ MDSCs (29). Notably, blocking A2BR decreases the number of MDSCs in the TMEs, thereby reducing levels of vascular endothelial growth factor (VEGF) and IL-10 (30). Moreover, MDSCs with high expression of CD39 and CD73 also shape the immunosuppressive microenvironments by directly suppressing T cells and NK cells, resulting in lower responsiveness to chemotherapy in patients with NSCLC (31).

## Targeting adenosine based on biomaterials for cancer immunotherapy

Given the active role of eADO in immune escape, the effectiveness of cancer immunotherapy is limited. Many Phase I/II clinical trials blocking the adenosine signaling pathway are already underway, mainly with drugs targeting the CD39-CD73-A2AR/A2BR axis (5). They mostly include CD39/CD73 inhibitors that prevent adenosine production and A2AR/A2BR inhibitors that block the binding to immune cells to achieve reversal of the immunosuppressive microenvironments and boost antitumor immunotherapy. However, these small molecule inhibitors or monoclonal antibodies have limitations, such as short circulating half-lives, poor tumor penetration, and lack of affinity for lymphocytes, which make them less efficient. Furthermore, due to the diversity of adenosine sources and the synergistic effect of adenosine with other immunosuppressive factors, it is unrealistic to alleviate tumor progression using a single adenosine signaling pathway blockade. Cancer patients are more likely to benefit from adenosine-targeted immunotherapy, chemotherapy, or other combination therapeutic strategies, and integrating these options to achieve optimal efficacy and safety is what biomaterials excel at as carriers. This section will focus on the top-down regulation of adenosine signaling pathways by biomaterial-based strategies and the integration of biomaterials in these strategies to characterize multiple therapeutic modalities (**Figure 2**).

### Inhibition of adenosine binding to receptors

Blocking the binding of adenosine to receptors has become an important area of cancer research due to its direct effect on tumor-infiltrating T cells and NK cells, among others. This is because adenosine binds to A2AR or A2BR on the surface of immune cells and activates intracellular immunosuppressive signaling, a process restricted by adenosine receptor inhibitors.

To enhance the delivery of adenosine receptor inhibitors, modified biomaterials are first designed to target specific tissues and immune cells for active transport. A common strategy is to modify molecules, moieties, or antibodies that target immune cells, using the homing of immune cells to localize nanoparticles to the target tissue (6, 7, 32) (**Table 1**). For example, Siriwon et al. reported the cross-linked multilamellar liposome functionalized to chimeric antigen receptor-engineered T (CAR-T) surface by maleimide (32). The maleimide head group successfully bound to the surface of sulfhydryl-rich T cells. When biomaterials encapsulated with A2AR inhibitors bound to the surface of CAR-T cells, they sustainably restored CAR-T cells that were dysfunctional in the TMEs, providing a layer of protection to improve the antitumor effect of adoptive cell therapy. In addition, Francis et al. reported enhanced immune checkpoint therapy in blocking non-redundant immunosuppressive pathways *via* a PD-1 antibody-conjugated nanoplatform that targets immune checkpoint-expressing lymphocytes and co-delivers encapsulated A2AR inhibitors and TGF-β inhibitors to cells residing in lymph nodes (6). This strategy for delivering A2AR inhibitors to T cells *via* biomaterials improved drug bioavailability and acted synergistically with immune checkpoint inhibitors to reverse T cell depletion in a non-redundant manner. Likewise, combining biomaterial-based A2AR inhibition with CAR-T treatment will consistently protect T cells from immunosuppressive modulation by adenosine in the TMEs.

**Table 1:** Biomaterial-based strategies to regulate the adenosine signaling pathways for cancer immunotherapy.

Target	Biomaterial	Combination therapy	Immunoregulation	Ref.
A2AR	Cross-linked multilamellar liposomal vesicle	SCH-58261 and CAR T-cell therapy	Increase the number of T cells located at the TMEs and the secretion of IFN-γ	(32)
	PD-1 antibody-poly(propylene sulfide) nanoparticle conjugate	SCH-58261, PD-1 antibody, and SB-431542 (TGF-β inhibitor)	Enhance trageting of T cells to promote the proliferation of T cells and the secretion of IFN-γ	(6)
	E-selectin-modified nitrilotriacetic acid-PEG-poly-(acrylamideco-acrylonitrile) polymer	SCH-58261 and DOX	Promote DC maturation, increase T cells and decrease Tregs, also increase the secretion of IL-2, IL-6, IL-12p70, IFN-γ, and TNF-α, and decrease IL-10	(7)
	TST, CPT-S-PEG, and AZD4635 co-assembled nanoparticle	AZD4635, camptothecin, and PTT	Improve the proportion of cytotoxic NK cells and T cells, and reduce MDSCs	(33)
	Photoactivatable semiconducting polymeric nanoantagonist	Vipadenant and PTT	Activate DCs, increase the proportion of CTLs and memory T cells, decrease the proportion of Tregs, and elevate the secretion of IL-1β, IL-6, TNF-α, and granzyme B, when decrease IL-10 and TGF-β	(34)
	Polydopamine nanoparticle	SCH-58261 and PTT	Enhance DC maturation, increase the cytoxicity of CTLs, promote M1 polarization, decrease the proporation of Tregs and MDSCs, also increase the secretion of IL-6, IL-12p40, IFN-γ, and TNF-α	(35)
	RGD-PEG-ss-PCL polymer micelle	SCH-58261, DOX, and R848 (TLR7/8 agonist)	Increase the proporation of CTLs and NK cells, and decrease the proporation of Tregs, with elevating IFN-γ and TNF-α, and declining TGF-β	(36)
CD73 or CD39	Polyethylene glycol-thioketal-DOX polymer	Anti-CD73 antibody, DOX, and PDT	Active DCs with increasing IL-6, IFN-γ, and TNF-α levels, and enhance memory T cells	(37)
	DSPE-PEG2000-pep liposome	CD39 inhibitor POM-1 and oxaliplatin	Augment memory T cells and reduce Tregs and M2 macrophages	(38)
	Chitosan-lactate nanoparticle	CD73 siRNA and DC vaccine	Suppress the accumulation of Tregs and MDSCs in the TMEs	(39)
	MnFe_2_O_4_-DCA nanocomposite	DCA	Synergize with DCA to alleviate hypoxia to downregulate CD39 and CD73 and increase the ratio of CD44^+^CD62L^-^ memory T cells	(8)
ADA	Alginate hydrogel	ADA, benzene-1,2,3-tricarboxylic (autophagy inducer), and DOX	Enhance the infiltration of CTLs and memory T cells in the TMEs and increase cytokines such as IL-2, IFN-γ, and TNF-α	(40)

RGD-PEG-ss-PCL, arginine-glycine-aspartic acid-poly(ethylene glycol)-ss-poly(ε-caprolactone).

Second, using biomaterials to transport adenosine receptor inhibitors to the tumor site rather than to a specific cell would enable a broader reversal of the immunosuppressive microenvironments. Modifications of internal responsiveness of acid or redox or external stimulation conditions of light or heat all reserve opportunities for precise delivery, temporal and spatial controlled release, and synergistic treatment of biomaterials. Wang et al. reported the nanoparticle formed by the co-assembly of a camptothecin prodrug (camptothecin-S-poly(ethylene glycol); CPT-S-PEG), a near-infrared light aggregation-induced emission molecule (TST), and the adenosine inhibitor AZD4635 (33). The limiting effect of camptothecin on TST was eliminated only in cancer cells where the single sulfur bond in CPT-S-PEG was oxidatively reduced. As a result, intact nanoparticle in circulation was degraded in tumor cells and induced immunogenic cell death (ICD), activating DCs and T cells. Moreover, the released AZD4635 bound to the A2AR on the surface of NK cells, T cells, and MDSCs, reversing the immunosuppressive effect of adenosine. The biomaterial system achieves multiple combination therapy of chemotherapy, immunotherapy, and photothermal therapy (PTT), which significantly inhibits tumor progression.

Central to this therapeutic strategy is the synergistic amplification of immunomodulatory effects of adenosine inhibitors with therapies that induce ICD. Either the biomaterial carries chemotherapeutic agents capable of inducing ICD, such as doxorubicin (DOX), oxaliplatin (OXA), and paclitaxel (PTX), or the biomaterial is linked to light-induced reactivity that triggers ICD (7, 34–36, 41) (**Table 1**). These treatments will directly kill tumor cells and induce the release of pro-inflammatory ATP and tumor antigens from tumor cells, subsequently initiating a specific antitumor immune response. Importantly, therapeutic strategies based on this principle would significantly increase the immunogenicity of tumors, reverse the immunosuppressive microenvironments, and enhance responsiveness to cancer immunotherapy.

### Reduction of adenosine in tumor microenvironments

Unlike the use of antagonizing adenosine receptors to block the downstream regulation of adenosine on immune cells, reducing adenosine levels in the TMEs hinders the immunosuppressive effects of adenosine at its source. Currently, strategies to disrupt adenosinergic axis focus on reducing the upstream production of adenosine and facilitating the downstream conversion of adenosine already produced in the TMEs (**Table 1**). In theory, reducing the adenosine content in the TMEs is a promising strategy to enhance antitumor immunotherapy.

CD39 and CD73 enzyme receptors convert ATP to adenosine *via* purine signaling, making them primary targets for regulating adenosine production. The effect of single-drug therapy is limited, and the combination of biomaterial-based therapeutic strategies with CD39 or CD73 inhibitors has achieved excellent tumor suppression in preclinical studies (5). For example, Jin et al. constructed cancer membrane-camouflaged, rose bengal and DOX co-loaded upconversion nanoparticle in combination of CD73 antibody for synergistic cancer therapy. This therapeutic strategy significantly controlled tumor progression and doubled the proportion of activated DCs in the microenvironments of distant tumors compared to the controls, promoting a systemic antitumor immune response in the 4T1 triple-negative breast cancer murine model (37).

However, inhibition of either A2AR or CD73 does not maintain immunostimulatory ATP levels in the TMEs. Inhibition of CD39 has the potential for a pluripotent effect, protecting released ATP from degradation by hindering the dephosphorylation of ATP to AMP in the adenosinergic axis while reducing eADO production. Fu et al. designed cationic liposome DSPE-PEG2000-pep formed by linking lipids 1,2-distearoyl-sn-glycero-3-phosphoethanolamine-*N*-[amino (polyethylene glycol)2000] (DSPE-PEG2000-NH_2_) and a small peptide (PLGLAG, pep). The pep in DSPE-PEG2000-pep broke in response to matrix metalloproteinase-2 and released loaded POM-1, a small molecule inhibitor targeting CD39, and ICD-inducing OXA (38). The negatively charged POM-1 adsorbed to the liposome surface *via* electrostatic interaction and detached and inhibited the adenosinergic pathway when bound to the higher affinity CD39 receptor in the TMEs. The synergistic effect of sustained ATP induction by chemotherapeutic agents oxaliplatin and CD39 inhibitors would sustain a high concentration of ATP in the TMEs to activate the immune response and increase the proportion of CD8^+^ T cells and the secretion of IFN-γ to promote antitumor immunotherapy.

In addition to the combination with CD39 or CD73 inhibitors to block the process of ATP hydrolysis, direct downregulation of CD39 and CD73 expression is also a meaningful way to inhibit adenosine production. Biomaterial-based strategies innovatively delivering specific siRNAs or alleviating hypoxia in the TMEs strongly reduce the adenosine content in the TMEs. Modulation-specific gene silencing is a straightforward approach. Gene vectors must have high gene transfection efficiency (i.e., easy internalization) and low cytotoxicity. As Hadjati and coworkers reported, highly biocompatible chitosan-lactide nanoparticle loaded with CD73 siRNA significantly reduced CD73 expression levels in tumor tissues of 4T1 breast cancer-bearing mice (39). Meanwhile, the nanoparticle reversed the adenosine-mediated immunosuppressive TMEs by synergistic treatment with the DC vaccine, reduced Tregs, MDSCs, and TAMs, and promoted CTL proliferation.

Moreover, relieving the hypoxic conditions of the TMEs is an indirect way to reduce the expression level of ATP-converting enzyme receptors. Warburg effect of tumor tissue and hypoxia caused by insufficient oxygen supply is one of the main features of the TMEs and is associated with immune escape (42). Although the mechanism is unclear, hypoxia has been shown to upregulate CD73 and CD39 expression in tumor cells (43). Taking advantage of this feature, Dai et al. designed MnFe_2_O_4_-dichloroacetic acid (MnFe_2_O_4_-DCA) nanocomposite to continuously supply oxygen to alleviate hypoxia while remodeling glycolytic metabolism (lactate production) to OXPHOS (ATP production) (8). MnFe_2_O_4_ nanoparticle oxidized glutathione and decomposed H_2_O_2_, releasing oxygen and hydroxyl radicals. Simultaneously, the delivered DCA effectively inhibited the expression of hypoxia-inducible factor 1α and remodeled glycolytic metabolism. The interplay of the two components downregulated the expression of CD39 and CD73 by 50% and 70%, respectively, significantly inhibiting ATP catabolism and reducing ADO levels in TMEs.

In addition to inhibiting adenosine production by enriching the ATP pool, promoting the conversion of immunosuppressive adenosine to immunoreactive substances is a novel strategy. Adenosine is converted to inosine by the catalysis of adenosine deaminase (ADA). Unlike immunosuppressive adenosine, inosine is an alternative energy source for effector T cells and supplements the energy supply of T cells in a glucose-deficient microenvironment to prevent T cell nutrient depletion (44). Therefore, synergistic transport of ADA with ICD-inducing drugs reduces the accumulation of adenosine in the TMEs and achieves cascade amplification of ATP-activated antitumor immunity (40). As Zhao et al. reported, the injectable hydrogel composed of sodium alginate, ADA, DOX, and autophagy inducer was constructed (40). Sodium alginate was chelated with calcium ions enriched in the TMEs to form an *in situ* hydrogel. The hydrogel acted as a drug reservoir in mice with sustained drug release and was effective for approximately twice as long as the free drugs, significantly inhibiting the progression of murine-derived B16F10 melanoma.

## Conclusion and prospects

Recent preclinical studies and clinical trials have provided compelling evidence for the immunomodulatory role of the adenosine signaling axis. Under physiological conditions, released ATP is followed by quenching through dephosphorylation of surface receptors, such as CD39 and CD73, and adenosine production to maintain anti-inflammatory immune homeostasis. However, this self-protective mechanism is exploited by tumors to cause immune escape.

Drugs, including inhibitors of A2AR, CD39, and CD73, that modulate the adenosine signaling axis have been developed and are in clinical trials. However, the problems of low single-drug utilization and poor efficacy are the bottlenecks that need to be overcome. Biomaterial-based delivery systems improve drug utilization by delivering drugs inside the tumor and distributing them within the TMEs for a longer period. With monotherapy, however, it is difficult to achieve complete eradication of tumors and removal of recurrent tumor foci. Combination therapy achieves real-time monitoring and control of new tumors by activating systemic and long-term memory immunity. The means of combination therapy (e.g., radiotherapy, chemotherapy, PTT, etc.) lead to tumor cell death and inhibition of tumor progression. However, the ensuing uncontrolled leakage of ATP and accumulation of adenosine within the TMEs induce a robust immunosuppressive effect. Biomaterial-based combination therapies integrate effective payloads, achieve sequential controlled and responsive release of drugs, and tailor definitive interventions to specific adenosine metabolic signaling profiles for optimal efficacy of immunotherapy. Meanwhile, these biomaterial-based strategies effectively balance extracellular ATP depletion, adenosine production, and ATP conversion, thereby maintaining high concentrations of pro-inflammatory ATP within the TMEs and inhibiting the immunosuppressive transduction of the adenosine signaling axis in immune cells in the TMEs.

However, studies targeting the adenosinergic pathway based on biomaterials are still lacking, and most of them only regulate a single target in the adenosine signaling axis. Given that various components of the adenosinergic pathway have redundant and non-redundant functions, it is essential to identify common targets for inhibitors of A2AR, CD39, and CD73. Furthermore, as tumor cells and various immune cells in the TMEs are affected by adenosine levels, the targeting of biomaterials is needed to identify the specific cell types that are regulated and the deep mechanisms downstream of them to exert optimal immunomodulatory effects to promote cancer immunotherapy.

## Author contributions

QW wrote the manuscript and made the illustration. LZ, NZ, ZC, HX, and JD reviewed and edited the manuscript. LZ, ZC, and HX provided the funding support. HX and JD designed and supervised the manuscript. All authors contributed to the article and approved the submitted version.

## Funding

This work was financially supported by the Science and Technology Development Program of Jilin Province (Grant No. 20210101230JC and 20200201353JC), the Science and Technology Research Project of Jilin Provincial Education Department (Grant No. JJKH20211199KJ), and the Special Project for Health Research Talents of Jilin Province (Grant No. 2021SCZ36).

## Acknowledgments

The authors appreciated the assistance from Changchun Institute of Applied Chemistry, Chinese Academy of Sciences, in terms of professional training.

## Conflict of interest

The authors declare that the research was conducted in the absence of any commercial or financial relationships that could be construed as a potential conflict of interest.

## Publisher’s note

All claims expressed in this article are solely those of the authors and do not necessarily represent those of their affiliated organizations, or those of the publisher, the editors and the reviewers. Any product that may be evaluated in this article, or claim that may be made by its manufacturer, is not guaranteed or endorsed by the publisher.
